# Fish intake and risk of heart failure: A meta-analysis of five prospective cohort studies

**DOI:** 10.3892/etm.2012.605

**Published:** 2012-06-08

**Authors:** LI-NA HOU, FEI LI, YOU ZHOU, SHI-HUAI NIE, LIANG SU, PING-AN CHEN, WAN-LONG TAN, DING-LI XU

**Affiliations:** 1 Departments of Cardiology and; 2 Urology, Nanfang Hospital, Southern Medical University, Guangzhou, Guangdong 510515, P.R. China;; 3 Minerva Foundation Institute for Medical Research, Biomedicum 2U, FI-00290 Helsinki, Finland;; 4 Department of Radiology, Zhongshan Ophthalmic Center of Sun Yat-sen University, Guangzhou, Guangdong 510060, P.R.China

**Keywords:** fish, nutrition, heart failure, meta-analysis

## Abstract

The findings on the association between fish intake and the risk of heart failure (HF) have been inconsistent. The purpose of this study was to clarify this potential association. We searched for relevant studies in the PubMed database through January 2012 and manually reviewed references. Five independent prospective cohort studies involving 5,273 cases and 144,917 participants were included. The summary relative risk estimates (SRRE) based on the highest compared with the lowest category of fish consumption were estimated by variance-based meta-analysis. In addition, we performed sensitivity and dose-response analyses to examine the association. Overall, an absence of an association between fish intake and HF was observed (SRRE=1.00; 95% CI, 0.81–1.24). However, fried fish intake positively associated with HF (SRRE=1.40; 95% CI, 1.22–1.61). In addition, dose-response analysis of fried fish suggested that each increment of six fried fish per month corresponded to a 37% increase of HF rate (RR=1.37; 95% CI, 1.20–1.56). In conclusion, our findings suggest that there is no significant association between fish intake and risk of HF, with the exception of a possible positive correlation with individuals comsuming fried fish, based on a limited number of studies. Future studies are required to confirm these findings.

## Introduction

Heart failure (HF), one of the most common reasons for hospitalization in the US Medicare population, remains a major threat to public health (1). It affected more than 5 million Americans in 2010 (2). Furthermore, it causes substantial mortality and morbidity. A previous study indicated that approximately 20% of people in the world will have HF at some point in their lifetime (3). Although diagnostic intensity and treatment is improving, prognosis is still poor (4). In addition, the rising incidence of HF is a cause for concern and there are few effective preventive measures against it (5). Therefore, feasible preventive measures of HF are of considerable clinical and public health importance (6).

Potential correlations between HF and different risk factors have been suggested (7). Smoking, age and diabetes are well-established risk factors of HF (8). Moreover, the associations between HF risk and exogenous factors including diet and lifestyle characteristics have been reported in numerous epidemiology studies (9,10). Certain studies report that high intakes of several specific types of fruits, vegetables and nutrients may decrease the risk of HF (11).

It is generally accepted that fish has excellent health benefits for humans (12). Fish is rich in the long-chain marine ω-3 polyunsaturated fatty acids (PUFAs), eicosapentaenoic acid (EPA) and docosahexaenoic acid (DHA), which may lower the cardiovascular disease risk by decreasing inflammation, oxidative stress and blood pressure, as well as improving cardiac and endothelial function (13,14). The relationship between fish intake and HF has received much attention since 1980. A few prospective cohort studies examined the potential correlation between fish intake and HF risk (15–19); however, their findings were controversial. Fish is one of the most common foods in the world. There is great interest in quantifying its independent association with HF incidence. To date, no quantitative assessment concerning the association has been conducted.

In the present study, we conducted the first meta-analysis to clarify the potential association between fish intake and risk of HF on the basis of findings from all published prospective cohort studies. Our aim was to estimate the relative risk of high intake versus low intake of fish. We also quantified the dose-response relationship between fish intake and HF risk.

## Materials and methods

### Literature search strategy

We conducted the present meta-analysis in accordance with Meta-analysis of Observational Studies in Epidemiology (MOOSE) guidelines (20). We performed a PubMed database search throughout January 2012 for relevant studies that reported the association between fish intake and risk of HF. The primary search included the following terms, diet, seafood, fish, heart failure or HF. The search focused on human studies, without a restriction on language. In addition, we reviewed the reference lists of all included articles to obtain relevant studies.

### Inclusion and exclusion criteria

Studies were included in the present meta-analysis if they met the following criteria; i) they should be prospective cohort studies in humans; ii) the primary outcome has been clearly defined as HF; iii) the study has examined the association between fish intake and HF risk from 1980 to January 2012; iv) the study has reported point estimates [i.e. relative risks (RR)s or odds ratios (ORs)] and measures of variability [i.e. 95% confidence intervals (CIs)] for the highest versus zero/lowest level of fish intake, or the studies provided sufficient information to estimate them. To avoid confusion, ‘fish’ in the present analysis included ‘fish’, ‘tuna fish’, ‘tuna and other fish’, ‘fried fish’ and ‘boiled fish’. A summary RR of fried or boiled fish would be calculated if they were presented individually in more than two studies. The exclusion criteria were i) duplicates; ii) no usable data reported; iii) cross-sectional, case-control and ecological analyses. We identified eligible articles for a full-text review following an initial screening by title or abstract.

### Data extraction

Two of the authors (L-N.H. and F.L.) independently extracted the information using a standardized data collection form from the selected studies. Any discrepancy was resolved by repeating the study review and discussion. The following information was recorded; name of the first author, year of publication, study location, study duration, follow-up time, number of cases, total number of participants, age range of participants, person-years of follow-up, number of exposed cases, categories of fish intake, the amount of fish intake for each category, RR or OR, the corresponding 95% CIs and adjusted confounding factors in the analysis. If one study reported multiple data sets, we used the results from the main multivariable model that included the most adjusted confounders.

We assessed the quality of each study by monitoring crucial components of the eligible studies; clear definition of participant characteristics, clear examination of exposure and outcome, study duration, sufficient duration of follow-up, person-years of follow-up, no selective loss during follow-up and control for potential confounding factors. If a study did not clearly mention one of these key points, we considered that it had not been performed, therefore it is likely that the reported characteristics were underestimated.

### Statistical analyses

We used the summary relative risk estimate (SRRE) for the highest compared with the lowest category of fish consumption. It should be noted that the lowest category included individuals who did not consume any fish. We used both the fixed- and random-effects method to estimate the association of HF and risk ratio of the highest category of fish consumption versus the lowest category. Statistical heterogeneity across studies was examined using the Q statistic (significant at P<0.10). The I^2^ statistic (values of 75, 50 and 25% were considered to represent high, medium and low heterogeneity, respectively) was also calculated to quantitatively measure the inconsistency across studies (21). Forest plots were constructed to assess the association between fish intake and HF risk.

Stratified analyses were conducted to investigate potential sources of heterogeneity, including geographical region, gender and cooking method of fish. In addition, we performed a sensitivity analysis to evaluate the influence of an individual study on the overall result. Each study was omitted in turn to assess the robustness of the results. A dose-response analysis was conducted based on the category data of fish intake, number of cases, person-years and logarithm of SRRE and its corresponding standard error. The eligible studies should provide sufficient information across at least three categories of exposure (22). Among the studies, we assigned a median of fish intake for each category. For the open-ended upper category of consumption, the amplitude was assumed the same as the previous one.

To examine whether publication bias affected the validity of the summary estimates, we applied Egger’s test and Begg’s method to evaluate the possible bias combined with a visual inspection of the funnel plot. Begg’s method is used to test the rank correction between the standardized effect size and the variances based on Kendall’s method (23). Egger’s test is a linear regression approach to measure the estimate divided by its standard error against the reciprocal of the standard error of the estimate (24). In other words, Egger’s method regresses the normalized effect size against precision. All statistical analyses were performed with STATA Statistical Software, version 11.0. P<0.05 was considered to indicate a statistically sigificant result, except where specifically noted.

## Results

### Literature search

**Figure 1:**
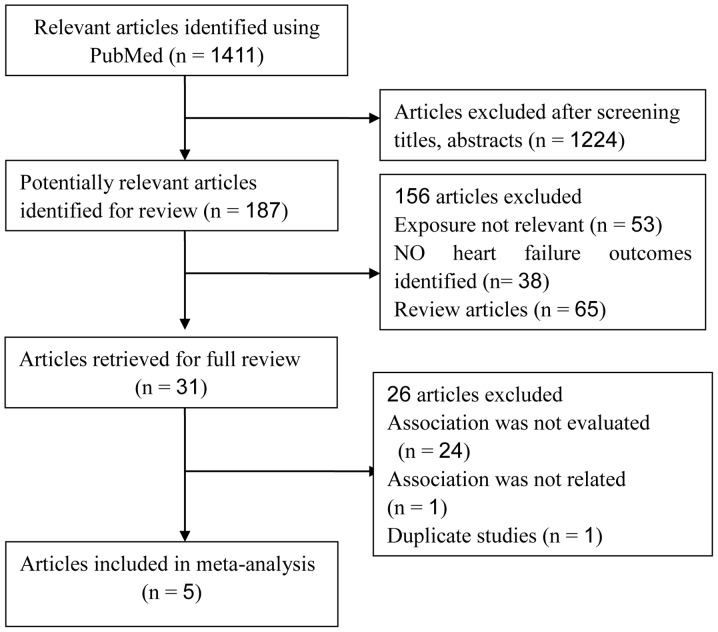
Flow chart of study selection.

Fig. 1 shows a flow chart of our selection process. A total of 1411 records were retrieved via a PubMed search. Of these, 1224 articles were excluded following an initial screen of abstracts and titles. Subsequently, 156 articles were excluded since they were review articles, did not have relevant exposure or no incidence of HF was identified. We identified 31 articles by full text review which evaluated the correlation between fish consumption and HF risk. Among the excluded 26 articles, one study was excluded as its participants overlapped with another study (25), a different study was not included due to the reported association with regard to fish intake and mortality from HF (26). The remaining 24 studies were excluded due to their effect sizes and the corresponding 95% confidence intervals had not been provided or could not be calculated due to insufficient information. Finally, seven data sets from five independent prospective cohort studies were included in our analysis. These studies were published between 1980 and January 2012.

### Study characteristics

The characteristics of the five included studies are listed in Table I. Of the five studies, two studies were conducted in the US (18,19), one in Sweden (16), one in The Netherlands (17) and one in nationwide clinical centers (15). The period of follow-up ranged from 9 to 13.3 years. Studies in our analysis used a Food Frequency Questionnaire (FFQ) based on self-report or interviewer-administered questionnaires to ascertain dietary information relating to fish intake, despite food items differing in the questionnaire across studies. In summary, five studies comprising 5,273 cases and 144,917 participants were included in our analysis.

### Fish intake and HF risk

**Figure 2:**
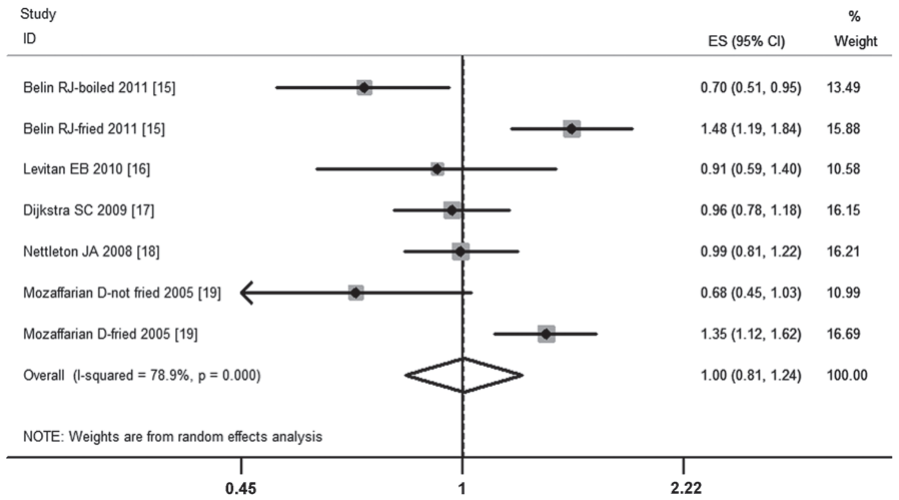
Meta-analysis of studies that examined fish intake and risk of heart failure. CI, confidence interval; ES, effect size.

Fig. 2 shows the pooled results from combing effect sizes for HF using the random-effects model. Overall, we found no significant association between fish intake and HF risk (SRRE=1.00; 95% CI, 0.81–1.24). Substantial heterogeneity was detected across studies (P-value for heterogeneity <0.0001, I^2^=78.9%; Fig. 2).

### Other correlations

**Figure 3:**
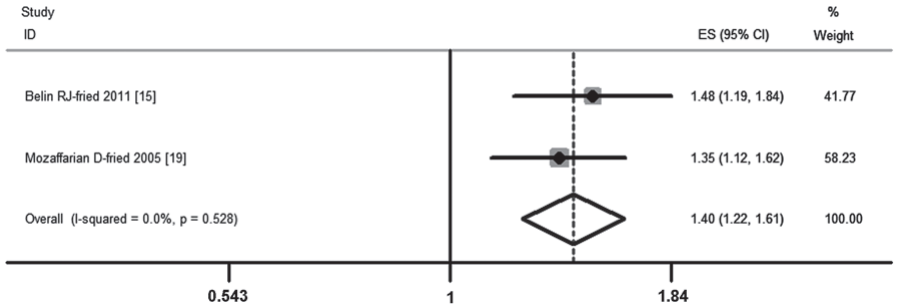
Meta-analysis of studies that examined fried fish intake and risk of heart failure. CI, confidence interval; ES, effect size.

When the studies were stratified by geographical region, no significant association was observed [USA (18,19): SRRE=1.01; 95% CI, 0.72–1.41; P-value for heterogeneity=0.004; I^2^=81.7%; Europe (16,17): SRRE=0.95; 95% CI, 0.79–1.15; P-value for heterogeneity=0.827; I^2^= 0%]. Notably, there was no variability across the studies conducted in Europe (P-value for heterogeneity= 0.827; I^2^= 0.0%) compared to the studies conducted in the US (P-value for heterogeneity=0.004; I^2^=81.7%). Two studies had only female participants (15,16). The SRRE of these two studies was 0.99 (95% CI, 0.60–1.65; P-value for heterogeneity<0.0001; I^2^=87.5%). In the cooking method subgroups we observed a significant positive association between fried fish consumption and HF (15,19). The risk of HF markedly increased by 40% on the basis of comparisons between the highest and lowest quartiles of fish intake (SRRE=1.40; 95% CI, 1.22–1.61), without any evidence of heterogeneity (P-value for heterogeneity= 0.528; I^2^=0%; Fig. 3).

### Sensitivity testing and publication bias

Further sensitivity testing via the exclusion of a single study at a time suggested that no single study influenced the overall results in our meta-analysis, with a narrow range from 0.93 (95% CI, 0.82–1.03; P-value for heterogeneity= 0.001) to 1.05 (95% CI, 0.95–1.15; P-value for heterogeneity=0.001).

Visual inspection of the funnel plot (not shown) did not suggest substantial asymmetry. There was no statistical evidence of publication bias based on the Begg’s rank correlation (P=0.548) and the Egger’s linear test (P=0.126).

### HR and fried fish

Dose-response analysis of two studies on fried fish consumption provide sufficient consistent evidence that the incidence of HF was elevated when fried fish consumption increased. Each increment of six fried fish per month corresponded to a 37% increase of HF rate (RR=1.37; 95% CI, 1.20–1.56). This was consistent with our combined results on fried fish consumption. We did not conduct the dose-response analysis of studies on overall fish intake as there was no connection to the risk of HF in our analysis.

## Discussion

The benefits of fish consumption are thought to be largely attributable to the antiarrhythmic activity of abundant PUFAs in fish (12–14). Epidemiological evidence suggests that the high intake of fish may reduce risks of stroke and coronary heart disease (CHD) (27,28). This raises great interest in whether fish intake has any relationship with HF, a type of cardiovascular disease.

Therefore, we conducted the first meta-analysis for clarification of the association between fish intake and HF risk. Five prospective cohort studies comprising 144,917 participants were included in our analysis. The combined results suggested no correlation between fish intake and HF incidence. The findings were similar for subgroups according to geographical region or gender. Interestingly, we found that a high level of fried fish consumption was associated with a 40% increased risk of HF.

Heterogeneity is a major concern in meta-analyses. A marked heterogeneity was observed across the included studies. This may be caused by variability among the study populations, follow-up period, analytical methodology, dietary assessment method and adjustment for confounding factors. Based on subgroups according to geographical region, gender, method of fish cooking, little heterogeneity was observed among studies conducted in Europe and studies that assessed fried fish individually. We were not able to analyze other subgroups due to the limited data. However, the results of sensitivity analyses were similar and robust, indicating that no single study considerably influenced the overall risk estimate between fish intake and HF. In addition, we observed no evidence of publication bias in our meta-analysis based on Egger’s test and Begg’s rank correction.

Our results compare favorably with the majority of studies included in our analysis, where it was reported that consuming fish was not associated with HF risk, whereas consumption of fried fish was associated with a 40% higher risk of HF. Dose-response analysis of fried fish consumption suggested that a 37% increased risk of HF was caused by an incremental increase of an average of six fried fish per month.

The underlying mechanism involved in the association between fried fish consumption and HF is uncertain. One possible cause is that the net effect of benefit versus risk of fried fish consumption may be detrimental (29). Although the method of frying does not decrease the absolute n-3 fatty acid level, frying adds other fatty acids from the frying oil and the procedure of cooking at high temperatures may add oxidation products, partially hydrogenated oils and trans-fatty acids (30–32). These products may cause the HF risk to increase. In addition, the association between fried fish intake and HF risk was partly related to other higher risk clinical and lifestyle factors. The higher fried fish consumption was markedly correlated with a lower fiber and higher fruit/vegetable intake (15,19). A higher fried fish consumption could also cause a higher prevalence of diabetes, atrial fibrillation, CAD, higher systolic blood pressure, higher body mass index, higher prevalence of smoking and higher calorie intake (15). Therefore, these associated risk factors may contribute to HF. Higher blood pressure, vascular resistance and cardiac wall motion abnormalities may be the potential physiological basis by which fried fish intake affects the risk of HF (33).

Notably, our study has several key strengths. This is the first meta-analysis to quantitatively assess the relationship between fish intake and HF. The analysis was based on five well-established prospective cohorts which had minimized recall and selection biases. The studies had large sample sizes and long term follow-up periods that enhanced the statistical power to estimate the overall association between fish intake and HF risk. Moreover, in order to control the bias, the included studies were adjusted for a wide range of potential confounding variables. In addition, our pooled analysis of five studies involving 144,917 participants was able to detect a more stable association and provide a more reliable estimation.

There were a number of limitations to our current meta-analysis when interpreting the results. First, although not suggested by the Begg’s rank correlation and the Egger’s linear test, potential bias may be involved considering that the tests for bias were likely to be underpowered. However, our sensitivity test showed the findings were robust. Second, substantial heterogeneity was observed among the studies, although we were able to reveal that geographic region is a major source of heterogeneity via subgroup analyses. Third, residual confounders always raise a major concern in the epidemiology studies. Although most studies included in our analysis had performed adjustments for a wide range of dietary and lifestyle variables, we could not exempt the possibility that other uncontrolled or unmeasured confounding factors play roles in the summary associations. Fourth, all the included studies in our analysis were prospective cohort studies. However, differences among follow-up period, dietary assessment method and measurement of HF end point, may hinder an estimate of the true effects of fish or fried fish consumption on HF risk. An additional limitation is that we were unable to explore potential differences of associations according to classification of HF. It remains unclear if findings may vary by subtype.

In summary, it is generally accepted that there is a great benefit of fish intake. Fish contain numerous essential nutrients which benefit healthy living. Therefore, fish is generally considered a healthy diet choice. However, our meta-analysis on the basis of 144,917 participants suggests no significant association between fish intake and HF risk and provides evidence that the incidence of HF might be significantly increased by consuming a high level of fried fish. Due to limited data, more studies are required to confirm the findings.

## Figures and Tables

**Table I:** Characteristics of the studies that analyzed fish consumption and heart failure.

First author, year (ref.)	Study location	Cases^a^/subjects	Follow-up (years)/period	Items	Analytical comparison (high vs. low intake)	RR (95% CI)/Trend P-value	Adjustments
Belin RJ, 2011 (15)	USA	1,858/84,493	10.0/1998–2008	Baked/broiled fish	≥5 times/week vs. <1 time/month	0.70 (0.51–0.95) 0.022	Age, smoking, BMI, ethnicity, education, physical activity, alcohol, hypertension, diabetes, atrial fibrillation, MI/coronary artery bypass graft/percutaneous transluminal coronary angioplasty, time-dependent MI, fiber, fruit/vegetable servings, saturated fat intake (%), DHA EPA (%), ALA (%), linoleic acid (%), fried food servings and sodium intake (mg)
				Fried fish	≥1 time/week vs. <1 time/month	1.48 (1.19–1.84) 0.005	
Levitan EB, 2010 (16)	Swedish	651/36,234	9.0/1998–2006	Fish	≥3 servings per week vs. 0	0.91 (0.59–1.40) 0.049	Age, smoking, BMI, total energy, education, physical activity, living alone, postmenopausal hormone use, alcohol intake, fiber intake, sodium intake, intake of red or processed meat, family history of myocardial infarction before 60 years, self-reported history of hypertension and high cholesterol
Dijkstra SC, 2009 (17)	The Netherlands	669/5,299	11.4/1990–2002	Fish	≥20 g/day vs. 0	0.96 (0.78–1.18) 0.39	Age, gender, smoking, BMI, energy, education, intake of alcohol, total fat, saturated fat, trans-fat and meat
Nettleton JA, 2008 (18)	USA	1,140/14,153	13.3/1987–1998	Fish	Yes vs. No	0.99 (0.81–1.22) NR	Age, gender, smoking, energy intake, race, education level, physical activity level, drinking and prevalent disease status
Mozaffarian D, 2005 (19)	USA	955/4,738	12/1989–2002	Not fried fish	≥5 times/week vs. <1 time/month	0.68 (0.45–1.03) 0.009	Age, gender, smoking, BMI, energy intake, race, enrollment site, education, diabetes, prevalent coronary heart disease, stroke/transient ischemic attack, fried fish or tuna/other fish intake, leisure-time physical activity, saturated fat, fruits, vegetables and alcohol intakes
				Fried fish	1–2 times/week vs. <1 time/month	1.35 (1.12–1.62) 0.005	

BMI, body mass index; NR, not reported.

a Number of cases of heart failure in the study. DHA, docosahexaenoic acid; EPA, eicosapentaenoic acid; ALA, α lipoic acid.
